# Respuesta a la carta al editor: “Consideraciones acerca del uso de glucómetros durante la prueba de tolerancia oral a la glucosa” (https://doi.org/10.1515/almed-2024-0108)

**DOI:** 10.1515/almed-2024-0148

**Published:** 2024-11-18

**Authors:** Estéfani Martínez Chávez, Blanca Fabre-Estremera, Marta Manzano Ocaña, Pilar Fernández-Calle, Antonio Buño Soto, Paloma Oliver

**Affiliations:** Servicio de Análisis Clínicos, Hospital Universitario La Paz, Madrid, España

Estimado Editor,

Nos gustaría expresar nuestro agradecimiento a Lavin-Gomez BA. y Guerra Ruíz​ AR [1]. por sus valiosos comentarios sobre nuestro artículo titulado “Uso de glucómetros durante la prueba de tolerancia oral a la glucosa en niños para el diagnóstico de prediabetes y diabetes” [2]. Apreciamos enormemente su interés en nuestro trabajo, así como las reflexiones que han compartido, las cuales contribuyen al enriquecimiento del debate científico en este campo. A continuación, respondemos a las consideraciones que han planteado.

**Figura 1:**
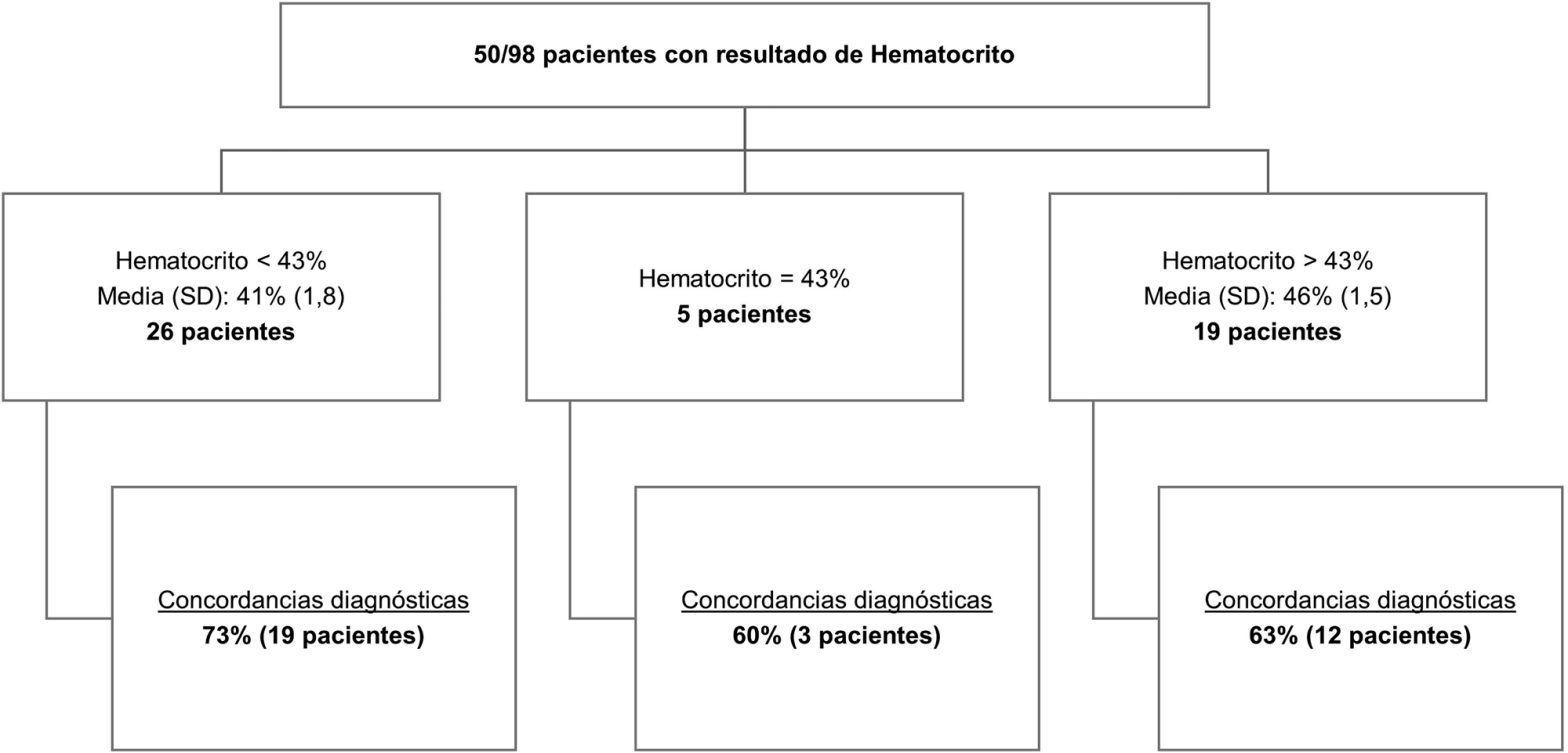
Concordancias diagnósticas entre POCT_ACI_ y el laboratorio central en función del hematocrito. Distribución de los 50 pacientes con resultados de hematocrito y análisis de concordancia diagnóstica entre POCT_ACI_ y el laboratorio central. Se agrupan en tres categorías: hematocrito inferior al 43 % (media 41 %, SD: 1.8, con una concordancia diagnóstica del 73 %), hematocrito igual a 43 % (concordancia diagnóstica del 60 %) y hematocrito superior al 43 % (media 46 %, SD: 1.5, con una concordancia diagnóstica del 63 %).

(1) Manejo preanalítico de las muestras.
  Agradecemos su observación y nos gustaría aclarar que, en nuestro centro se utilizan tubos de suero con gel durante la prueba de tolerancia oral a la glucosa. La Unidad de Diabetes dispone de una centrífuga para minimizar el efecto de la glucólisis *in vitro*. Reconocemos en el artículo que una de las limitaciones del estudio fue la posible influencia de la glucólisis *in vitro* en las muestras en ayunas. Gracias a la realización del estudio, pudimos optimizar el protocolo para garantizar que las muestras en ayunas se centrifuguen a los 20 minutos tras la extracción.
(2) Correlación entre las determinaciones de glucosa.
  Durante la comparación de métodos en el estudio, las diferencias observadas entre los valores obtenidos con los glucómetros POCT_ACI_ (Accu-Chek^®^ Inform-II, Roche Diagnostics, Basilea, Suiza) y los de nuestro laboratorio central (Atellica^®^Solution-CH; Siemens Healthineers, Erlangen, Alemania) no superaron nuestras especificaciones de calidad en las concentraciones de glucosa de 100, 125, 140 y 200 mg/dL. Por esta razón, consideramos que estas diferencias no eran clínicamente significativas. No obstante, decidimos examinar la correlación diagnóstica debido a la importante repercusión clínica que podría implicar una discrepancia en los diagnósticos.
  Con los resultados obtenidos coincidimos en que se observa una tendencia en la que las diferencias entre los resultados del POCT_ACI_ y el laboratorio aumentan a medida que se incrementa la concentración de glucosa, siendo los valores medidos con el glucómetro inferiores a los obtenidos en el laboratorio central a niveles altos. El diseño del estudio no nos permitió identificar la causa de por qué los resultados del glucómetro fueron más bajos en los tiempos de 30, 60 y 120 minutos en comparación con los resultados del laboratorio. Futuras investigaciones que controlen otras variables podrían ayudar a explicar estas diferencias observadas.
(3) Interferencia del hematocrito en la medición de glucosa.
  Los glucómetros que utilizamos en nuestro estudio, POCT_ACI_ y POCT_ACP_ (Accu-Chek^®^ Performa, Roche Diagnostics, Basilea, Suiza) no miden el hematocrito. Dado el interés en explorar esta variable, hemos analizado los valores de hematocrito de nuestra población de estudio. De los 98 pacientes incluidos, 50 tenían hemograma en la misma solicitud de laboratorio. El 68 % (34) presentaron concordancias diagnósticas entre POCT_ACI_ y el laboratorio central (ver Figura 1).

En el análisis individual de los casos con discrepancias, observamos que en 3 de los 4 pacientes con hematocrito superior al 43 % (rango 44–49 %), la discrepancia en el diagnóstico podría haber tenido un impacto clínico. En todos estos casos, los resultados obtenidos por el glucómetro fueron inferiores a los del laboratorio central. Por otro lado, de los 5 pacientes con hematocritos inferiores al 43 % (rango 36–42 %), en 2 casos la discrepancia en el diagnóstico podría haber tenido un impacto clínico. En ambos casos, los resultados del glucómetro también fueron inferiores a los del laboratorio (ver Tabla 1). Por lo tanto, cuando el hematocrito fue inferior al 43 %, no observamos la relación inversa entre este y la glucosa, tal y como describe la literatura [3].

En nuestra población de estudio, el glucómetro tendió a infradiagnosticar prediabetes o diabetes, a excepción de los casos de alteración de la glucemia en ayunas donde la discrepancia probablemente se debió a la glucólisis *in vitro* en las muestras en ayunas. En nuestros resultados, no observamos una clara influencia del hematocrito.

Compartimos la opinión de que futuros dispositivos con capacidad para medir el hematocrito simultáneamente podrían ofrecer lecturas más precisas y reducir las variaciones observadas, abriendo nuevas oportunidades para investigar si estos dispositivos pudiesen ser utilizados con fines diagnósticos en un futuro próximo. Agradecemos de nuevo a Lavin-Gomez BA. y Guerra Ruíz AR. por sus valiosos comentarios, que enriquecen el debate científico sobre el uso de glucómetros en entornos clínicos.

**Tabla 1:** Discrepancia diagnóstica entre POCT_ACI_ y el laboratorio central.

POCT_ACI_	Laboratorio central	Impacto clínico	Hematocrito, %
AGA	Normal	Sin impacto	36
AGA	Normal		40
AGA	Normal		42
AGA	Normal		43
AGA	Normal		?
AGA	Normal		?
AGA	Normal		?
Normal	ATG		47
AGA	AGA+ATG		?
ATG	Diabetes		?
Normal	ATG	Leve	41
ATG	Diabetes		44
ATG	Diabetes		?
Normal	ATG	Grave	48
Normal	ATG		?
GAA+ATG	Diabetes		47
ATG	Diabetes		41

AGA, alteración de la glucemia en ayunas; ATG, alteración de la tolerancia de glucosa; POCT_ACI_, glucómetro conectado.
